# The exon 12‐containing LHX6 isoforms promote cervical cancer cell proliferation by regulating the MAPK signaling pathway

**DOI:** 10.1002/cam4.4734

**Published:** 2022-04-05

**Authors:** Ling Wang, Ying Zhou, Canhui Cao, Shitong Lin, Wenhua Zhi, Danya Zhang, Jie Li, Rui Wei, Guiying Jiang, Hanjie Xu, Xueqian Wang, Ling Xi, Peng Wu

**Affiliations:** ^1^ Cancer Biology Research Center (Key Laboratory of the Ministry of Education) Tongji Hospital, Tongji Medical College, Huazhong University of Science and Technology Wuhan Hubei China; ^2^ Department of Gynecologic Oncology Tongji Hospital, Tongji Medical College, Huazhong University of Science and Technology Wuhan Hubei China; ^3^ Center for Reproductive Medicine, Department of Obstetrics and Gynecology Peking University Shenzhen Hospital Shenzhen Guangdong China

**Keywords:** cell proliferation, cervical cancer, LHX6 isoforms, MAPK signaling pathway

## Abstract

LIM homeobox 6 (LHX6) has been reported to be downregulated and inhibits cell proliferation in various cancers. Alternative splicing of LHX6 leads to six annotated isoforms, which can be found in the NCBI database. However, the expression patterns and potential roles of these isoforms remain poorly characterized in cervical cancer. Here, we demonstrated that the LHX6 isoforms containing exon 12 (LHX6^EX(+12)^ group) and isoforms lacking exon 12 (LHX6^EX(–12)^ group) were differentially expressed in cervical tissue by qRT‐PCR. The mRNA expression level of LHX6^EX(+12)^ group was higher than that of LHX6^EX(−12)^ group in cervical cancer tissue. Knockdown of LHX6^EX(+12)^ group and all LHX6 isoforms (LHX6^All^ group) inhibited cell growth, increased cell apoptosis, and induced cell cycle arrest from G0/G1 phase to S phase in vitro. Consistently, overexpression of the LHX6^EX(+12)^ group promoted cervical cancer cell proliferation in vitro. In contrast, no significant differences in cell proliferation were found between LHX6^EX(−12)^ isoform knockdown group and its control. RNA‐sequencing suggested that the LHX6^EX(+12)^ isoform group might exert its cancer‐promoting effects in cervical cancer via regulating MAPK signaling pathway. Downregulation of the LHX6^EX(+12)^ group significantly suppressed the phosphorylation of MRK, ERK, JNK, and P38 at the protein level. We also identified some unique biological processes and signaling pathways in which each isoform group might be involved. In summary, our results indicated that LHX6^EX(+12)^ isoform group was the dominant oncogenic type of LHX6 in cervical cancer, which may be a new biomarker and a potential precise therapeutic target for cervical cancer in the future.

## Graphical abstract

In this work, we evaluated the expression patterns and potential roles of different LHX6 isoforms in cervical cancer. Our study demonstrated that LHX6 isoforms containing exon 12 (LHX6^EX(+12)^) and isoforms lacking exon 12 (LHX6^EX(^^–^^12)^) were differentially expressed in cervical tissue. And LHX6^EX(+12)^ isoform group was the predominant oncogenic subtype of LHX6 in cervical cancer, which could be a new biomarker and a potential precise therapeutic target for cervical cancer in the future.

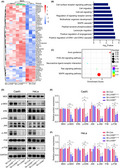

## 1 INTRODUCTION

Cervical cancer remains ranked fourth in both morbidity and mortality among females worldwide with 569,847 new cases and 311,365 deaths in 2018, even though much progress has been made in the detection and treatment over the past decades.^1^ And the 5‐year survival rate of patients at an early stage is over 90%, but this rate drops to 50% or less for advanced cervical cancer.^2^, ^3^ Hence, it is necessary to discover novel biomarkers and therapeutic targets to improve early diagnosis and effective treatment for cervical cancer.

Persistent infection with high‐risk human papillomavirus (HPV) genotypes is considered as the major cause of cervical cancer.^4^, ^5^, ^6^ HPV infection may induce genomic alterations, such as copy number variation, DNA methylation, and somatic DNA mutation, which eventually contribute to cervical carcinogenesis.^7^, ^8^ In addition to the abnormalities at transcriptome and epigenetic levels, recent studies have indicated that dysregulation of posttranscriptional splicing variants (alternative splicing) may also be associated with cervical cancer development and progression.^9^, ^10^, ^11^ It has been demonstrated that alternative splicing events are observed more often in tumors than in normal tissue samples.^12^ Abnormality of alternative pre‐mRNA splicing regulation can affect cell proliferation, cell apoptosis, cell metabolism, proto‐oncogenes activation, cell invasion and metastasis, and angiogenesis.^13^, ^14^, ^15^ More importantly, due to the abnormal alterations of alterative splicing, different transcripts from a single gene can play various roles in cancer initiation and progression, thus providing a potentially rich source of novel biomarkers, therapeutic targets, and guidance for targeted therapy by oncogenic isoform management.^16^, ^17^, ^18^, ^19^ Therefore, exploring the roles of different transcripts from the mother gene in cervical cancer may provide a new way for the identification of novel cervical cancer biomarkers and precise therapeutic targets for better treatment.

Human LIM homeobox 6 (LHX6), a member of a large family that contains the LIM domain, is a transcription factor involved in embryogenesis and head development.^20^ It has been reported that LHX6 expression is decreased in lung cancer,^21^ head and neck cancer,^22^ breast cancer,^23^ and liver cancer^24^ through epigenetic inactivation, indicating that it could be a sensitive biomarker for that cancer. And most studies indicate that LHX6 may inhibit cancer cell proliferation, migration, and invasion via repression of the Wnt/β‐catenin or P53 signaling pathway, suggesting its potential role as a tumor suppressor in multiple cancer types.^24^, ^25^, ^26^ Previously, we identified a highly frequent (>10%) chimeric RNA named LHX6‐NDUFA8 which was detected exclusively in cervical cancer tissues and Pap smears, but not in normal cervical tissues, supporting its potential as an early molecular biomarker for cervical cancer.^27^ These findings drove us to conduct further exploration on the roles of LHX6 and its isoforms in cervical cancer. Human LHX6 is located on chromosome 9q33.2 and is comprised of 14 exons. Six annotated LHX6 splice variants are listed in the National Center for Biotechnology and Information Database (NCBI, https://www.ncbi.nlm.nih.gov/gene/26468). However, we found that few studies had reported the expression patterns and biological roles of different LHX6 isoforms in cervical cancer.

Hence, this study aimed to investigate the precise expression patterns and biological functions of different LHX6 isoforms in cervical cancer, expecting to find a new biomarker and therapeutic target for it. In this work, we divided these isoforms into the LHX6^EX(+12)^ group (including isoforms 1, 4, and 5) and the LHX6^EX(−12)^ group (including isoforms 2, 3, and 6) according to whether they contained exon 12 or not. We detected, for the first time, the mRNA expression level of two different LHX6 isoform groups in cervical cancer and normal cervical samples by qRT‐PCR, and analyzed their clinical significance. To further explore the biological roles of two LHX6 isoform groups, knockdown and regain of functional experiments were conducted and we identified differences in the biological roles of the LHX6^EX(+12)^ isoform group and LHX6^EX(−12)^ group in cervical cancer cells. Furthermore, RNA sequencing and bioinformatics analysis were performed to explore the potential downstream signaling pathways and then were further confirmed by western blot assay.

## 2 MATERIALS AND METHODS

### 2.1 Clinical samples

Fifty‐nine cervical cancer tissue samples and 21 normal cervix tissue samples were obtained from the Biobank of Patients with Gynecologic Neoplasms of Tongji Hospital in Wuhan, China. Tissue sample information was presented in Table S1. These specimens were stored in −80°C until use. The study was approved by the Clinical Trial Ethics Committee of Huazhong University of Science and Technology.

### 2.2 Cell culture

Cervical cancer cell lines CasKi, SiHa, and HeLa were purchased from American Type Culture Collection and authenticated at China Center for Type Culture Collection (Wu Han University, China). Cells were cultured in Dulbecco's Modified Eagle Medium (DMEM, Gibco) containing 10% fetal bovine Serum (Gibco) and 1% penicillin–streptomycin mixture (Servicebio). Cells were kept in an incubator containing 5% CO_2_ atmosphere at 37°C. All the cells were tested to be free of mycoplasma contamination by a one‐step rapid mycoplasma detection kit (40612ES25, Yeasen).

### 2.3 RNA isolation and quantitative real‐time polymerase chain reaction (qRT‐PCR)

The total RNA of tissue specimens and cells were extracted by Trizol reagent (TaKaRa) following the instructions of the manufacturer. Isolated RNA of 1 μg was reversely transcribed to cDNA using HiScript II qRT SuperMix (R223‐01‐AC, Vazyme) in a final volume of 20 μl. Then qRT‐PCR was conducted to detect the gene expression level using iTaq™ Universal SYBR® Green Supermix (172–5124, Bio‐Rad) and EEF1A1 was used as an internal reference gene. The specific primer sequences were displayed: LHX6^EX(−12)^ group (Forward, 5′‐GGCTACATTGAGAGTCATCCTTT‐3′; Reverse, 5′‐GGTTCTCAGCAGGAGAGGGA‐3′); LHX6^EX(+12)^ group (Forward, 5′‐CCTGCACG GCTACATTGAGA‐3′; Reverse, 5′‐AGGATGACCTTCTCACCCCG‐3′); EEF1A1 (Forward, 5′‐TGTCGTCATTGGACACGTAGA‐3′; Reverse, 5′‐ACGCTCAGCTTT CAGTTTATCC‐3′). The results were calculated using the delta–delta‐Ct (ddCt) or the delta‐Ct(dCt) algorithm. All primers were synthesized by Tsingke Biotechnology (Beijing, China).

### 2.4 Transfection of lentivirus

Short hairpin RNA (shRNA) lentiviral particles were cloned into hU6‐MCS‐Ubiquitin‐firefly_Luciferase‐IRES‐puromycin vector to construct the LHX6 knockdown isoforms. The shRNA sequences were shown: Sh‐LHX6^EX(−12)^ group (5′‐TGAGAGTCATCCTTTTTCA‐3′); Sh‐LHX6^EX(+12)^ group (5′‐CCACCTCAAAGCCGATATG‐3′); Sh‐LHX6^All^ group (5′‐CCATCCTGCTACAACTCTT‐3′). For the establishment of LHX6 overexpressed cell lines, coding area sequence of LHX6 transcript variant 4 (NM_001242334) was cloned into Ubi‐MCS‐firefly_Luciferase‐IRES‐Puromycin vector. All the lentivirus were constructed by Genechem (Shanghai, China) and transfected cells according to the manufacturer's instructions.

### 2.5 Cell proliferation assay

Cell Counting Kit‐8 (CCK‐8), EdU incorporation assay, and colony formation assay were used to detect cell proliferation. Cells in the logarithmic growth phase were collected and counted. Cells were seeded at a density of 3000 cells per well into 96‐well plates in triplicate and cultured for 5 days. Cell culture medium was replaced every 2 days. The cell viability was assessed using a CCK‐8 Kit (Dojindo) according to the manufacturer's guidelines. In the following 5 days after the cells were seeded, the optical density at 450 nm (OD450) was measured using a microplate reader (SpectraMax ABS Plus) every day. For EdU incorporation assay, a total of 1 × 10^5^ cells were seeded in 24‐well plates and cultured for 24 h, then cell proliferation was examined by an EdU reagent Kit (RiboBio) following the instructions. Finally, the cells were photographed under an inverted fluorescence microscope (Olympus) and we set a uniform exposure time for the same fluorescence indicator. EdU positive rate = (EdU positive cell count/Hoechst positive cell count) × 100%. In the cell colony formation assay, the LHX6 isoform knockdown group and its control group cells were seeded in 6‐well plates at a density of 800 cells per well, and 400 cells per well were seeded in 6‐well plates for the LHX6^EX(+12)^ isoform‐overexpressed group and its control. Then the cells were incubated for 10 days. The clones were subsequently fixed with 4% paraformaldehyde for 25 min and stained with 0.5% crystal violet for 15 min, finally photographed after several washing. The number of cell clones (>50 cells/colony) were counted.

### 2.6 Cell apoptosis assay

A total of 5 × 10^5^ cells were seeded each well of 6‐well plates and then cultured with a complete medium for 48 h. Then the cells were collected by trypsinization and treated with FITC Annexin V Apoptosis Detection Kit I (556547, BD Pharmingen) according to the manufacturer's instructions. Flow cytometry (Beckman Coulter) was used to detect cell apoptosis. The apoptosis rate included the early and late apoptosis cell counts.

### 2.7 Cell cycle assay

Cells in the logarithmic growth phase were harvested and counted under sterile conditions. The cells were seeded at a density of 2 × 10^5^ cells per well into 6‐well plates and cultured with a complete medium for 48 h. Then the cells were collected and fixed with cold 70% ethanol overnight at −20°C, after washed two times with phosphate buffer saline (PBS), the cells were incubated with a mixture including 10 μg/ml propidium iodide (PI, Sigma‐Aldrich) and 100 μg/ml RNase A (Thermo Fisher Scientific) for 30 min at room temperature in the dark. After washing and filtering, cell cycle analysis was performed using flow cytometers.

### 2.8 Western blot assay

A total of 5 × 10^5^ cells were seeded each well of 6‐well plates and then cultured with a complete medium for 48 h. The cells were harvested and then lysed by radioimmunoprecipitation assay (RIPA) lysis buffer (Servicebio) containing protease inhibitor cocktail (Servicebio) for 20 min on ice. Protein concentration was measured by the Coomassie brilliant blue G‐250 staining method. Then the protein was boiled and denatured*,* and 25 μg of each sample was separated by 10% SDS–PAGE electrophoresis. Afterwards the separated protein was transferred onto a polyvinylidene difluoride (PVDF) membrane. Then the membranes were blocked with 5% albumin bovine V (Servicebio) for 1 h at room temperature and incubated with primary antibodies at 4°C overnight. Primary antibodies used were shown as follows: LHX6 (1:1000–1:2000, GTX55690, genetex), p53 (1:500–1:1000, 10442‐1‐AP, proteintech), p21 (1:500–1:1000, 10355‐1‐AP, proteintech), cyclinD1 (1:1000, A19038, abclonal), cleaved‐PARP (1:800, ab32064, abcam), GAPDH (1:8000, 60004‐1‐Ig, proteintech), MEK (1:1000, A19565, abclonal), p‐MEK (1:1000, AP1021, abclonal), ERK (1:1000, A4782, abclonal), p‐ERK (1:1000, AP0472, abclonal), P38 (1:1000, A4771, abclonal), p‐P38 (1:1000, AP0526, abclonal), JNK (1:1000, A4867, abclonal), p‐JNK (1:1000, AP0276, abclonal). The membranes were then washed with tris‐buffered saline containing tween‐20 (TBST, pH 7.5) and incubated with secondary antibody (1:10000, A21020, abbkine) for 1 h at 37°C. Finally, the protein was detected using a multi‐mode chemiluminescence system (Bio‐rad). The densitometric analyses of protein bands were measured by ImageJ software.

### 2.9 RNA‐sequencing analysis

Cells in the logarithmic growth phase were collected and counted under sterile condition. Cells were seeded into each well of 6‐well plates at a density of 5 × 10^5^ cells and cultured with a complete medium for 48 h. Then the cells were prepared for RNA‐sequencing (RNA‐seq). In brief, total RNA of cells were extracted by Trizol regent. The RNA was fragmented and transcribed to cDNA, then amplified to construct a cDNA library, afterwards subjected to sequencing on Illumina HiSeqTM 2500 sequencing machine. The acquired data were mapped to the human genome 38 and gene expression was calculated using Fragments Per KB Per Million Reads (FPKM) method. Deseq software was used to acquire differentially expressed genes. Then the Gene Ontology (GO) database and Kyoto Encyclopedia of Genes and Genomes (KEGG) database were used for relevant bioinformatic analysis. RNA‐seq was completed by Oebiotech (Shanghai, China).

### 2.10 Statistical analysis

Flow cytometry data were processed by FlowJo software (version 10). The statistical analysis was completed by GraphPad Prism software (version 8.0.2). The expression level of LHX6 isoforms in clinical samples was calculated by the chi‐squared test or Mann–Whitney *U* test and described as median. Other data were calculated by unpaired *t*‐test or ANOVA and described as mean ± standard deviation (SD). All the experiments were performed in triplicate. Differences were considered to be statistically significant at *p* < 0.05.

## 3 RESULTS

### 3.1 Distinct expression patterns of LHX6 isoforms in cervical cancer samples

**FIGURE 1:**
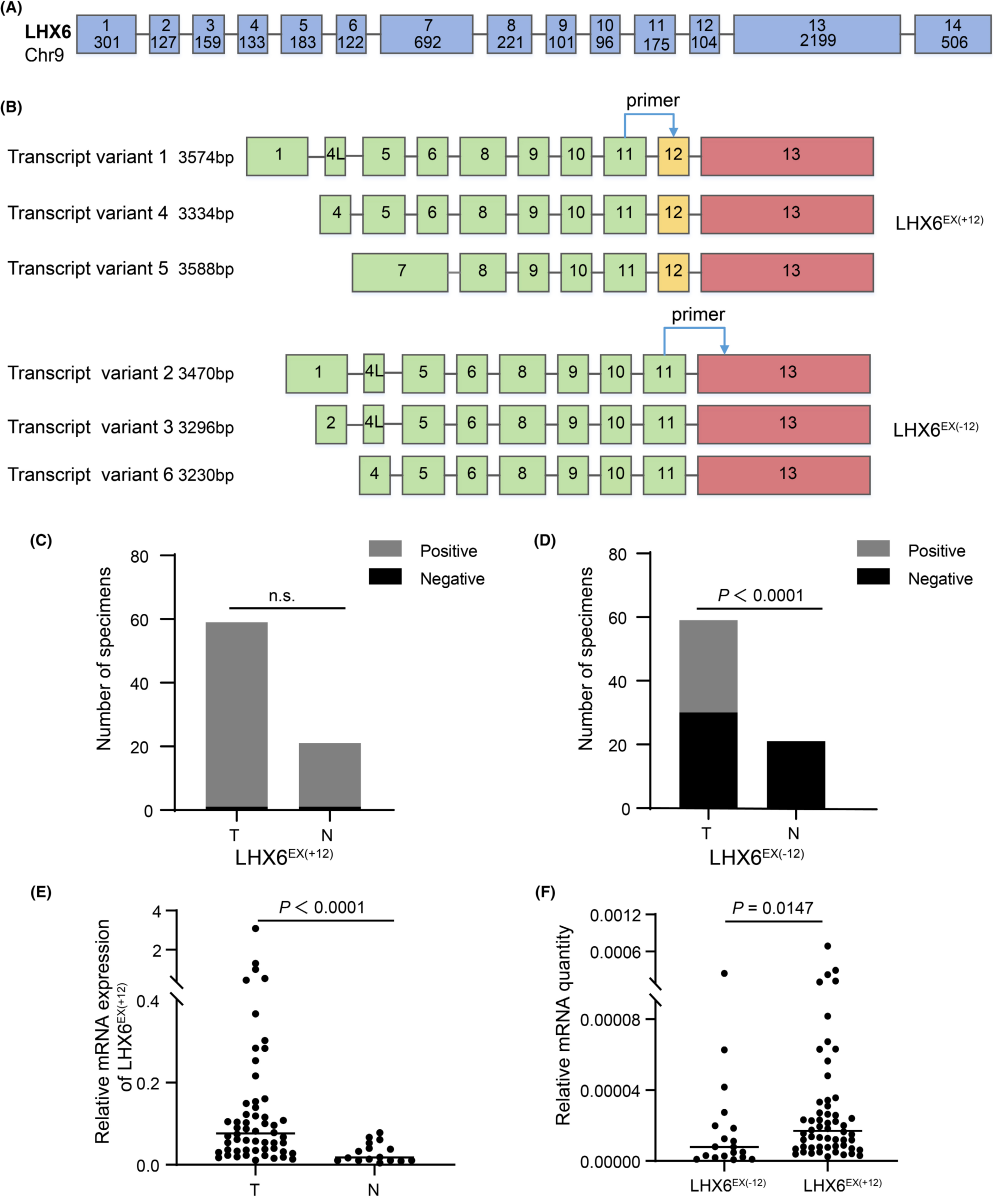
Model description and mRNA expression of LHX6 splice variants in cervical tissue. (A, B) Structures of LHX6 and its six annotated splice variants were depicted in the cartoon diagram. Boxes represent exons. The size of exons were indicated within the boxes. The location of arrows represented the position of specific primers designed. The exon 4 L indicated that this exon contained part length of exon 4 (the contained length was 72 bp). (C, D) The detection rate of LHX6^EX(+12)^ and LHX6^EX(−12)^ group in cervical cancer tissues (T) and normal cervical samples (N). (E) Relative mRNA expression level of LHX6^EX(+12)^ group in T and N. (F) Relative mRNA quantity of LHX6^EX(−12)^ and LHX6^EX(+12)^ group in T. The data were presented as median. n.s. indicated there was no significant statistical difference

As shown in Figure 1A–B, LHX6 contained 14 exons, with isoforms 1, 4, and 5 containing exon 12 (i.e., LHX6^EX(+12)^ group) and isoforms 2, 3, and 6 lacking exon 12 (i.e., LHX6^EX(−12)^ group). The LHX6^All^ group represented all six annotated isoforms. To clarify the expression patterns of LHX6 splice variants in cervical cancer, qRT‐PCR was first conducted in normal cervical (NC) epithelium (*n* = 21) and cervical cancer (CC) samples (*n* = 59). Results showed that LHX6^EX(+12)^ group was detected in both NC and CC tissues, with the positive rate of 95.24% and 98.31%, respectively (Figure 1C and Table 1). In contrast, the LHX6^EX(−12)^ group was only detected in 49.15% of CC samples (Figure 1D and Table 2, *p* < 0.0001). Further analysis revealed that the mRNA expression level of LHX6^EX(+12)^ group was increased in the CC samples compared with that in the normal tissue (Figure 1E, *p* < 0.0001). As shown in Figure 1F, the relative mRNA quantity of LHX6^EX(+12)^ group was higher than that of the LHX6^EX(−12)^ group in cancer tissues (*p* = 0.0147), indicating that the LHX6^EX(+12)^ group was the dominant component of LHX6 in cervical cancer. However, no significant correlations were found between the expression levels of the two isoform groups and clinicopathological parameters such as lymph node metastasis, histological differentiation, FIGO stage, and pathology type (Table 1 and Table 2).

**TABLE 1:** The correlation between LHX6^EX(+12)^ expression level and clinical parameters of cervical cancer tissue samples

Variable	No. of patients	LHX6^EX(+12)^ gene expression				Median of LHX6^EX(+12)^relative expression	*p* value
		Negative		Positive			
		No.	%	No.	%		
Cervical cancer patients							
Lymph node metastasis							
Negative	39	1	2.56	38	97.44	0.0672	0.2417
Positive	20	0	0.00	20	100.00	0.0818	
Differentiation							
Well or Moderately	29	1	3.45	28	96.55	0.0827	0.8693
Poorly	23	0	0.00	23	100.00	0.0857	
FIGO Stage							
IA‐IB	29	1	3.45	28	96.55	0.0889	0.5848
IIA‐III	30	0	0.00	30	100.00	0.0658	
Pathology							
Squamous carcinoma	48	0	0.00	48	100.00	0.0800	0.1533
Adenocarcinoma	8	1	12.50	7	87.50	0.0358	
Normal cervical tissues versus Cancer							
Normal cervical tissue	21	1	4.76	20	95.24	0.0175	**<0.0001**
Cervical cancer	59	1	1.69	58	98.31	0.0763	

*Note*: *p* value: calculated by Mann–Whitney *U* test.

FIGO: International Federation of Gynecology and Obstetrics.

**TABLE 2:** The correlation between LHX6^EX(−12)^ expression level and clinical parameters of cervical cancer tissue samples

Variable	No. of patients	LHX6^EX(−12)^ gene expression					Median of LHX6^EX(−12)^ relative expression	^b^ *p* value
		Negative		Positive		^a^ *p* value		
		No.	%	No.	%			
Cervical cancer patients								
Lymph node metastasis								
Negative	39	20	51.28	19	48.72	0.9257	0.1167	0.0874
Positive	20	10	50.00	10	50.00		0.5693	
Differentiation								
Well or Moderately	29	12	41.38	17	58.62	0.4350	0.1924	0.9661
Poorly	23	12	52.17	11	47.83		0.1194	
FIGO Stage								
IA‐IB	29	12	41.38	17	58.62	0.1526	0.0503	0.0789
IIA‐III	30	18	60.00	12	40.00		0.2847	
Pathology								
Squamous carcinoma	48	23	47.92	25	52.08	0.2996		
Adenocarcinoma	8	6	75.00	2	25.00			
Normal cervical tissues versus Cancer								
Normal cervical tissue	21	21	100.00	0	0.00	**<0.0001**		
Cervical cancer	59	30	50.85	29	49.15			

FIGO: International Federation of Gynecology and Obstetrics. The bold *p* values indicated the difference between the two groups of data was statistically significant.

^a^ *p* value: calculated by chi‐squared test.

^b^ *p* value: calculated by Mann–Whitney *U* test.

### 3.2 Downregulation of LHX6^EX^ ^(+12)^ and LHX6^All^ group suppressed cell proliferation, induced cell apoptosis, and arrested cell cycle in cervical cancer cells in vitro

**FIGURE 2:**
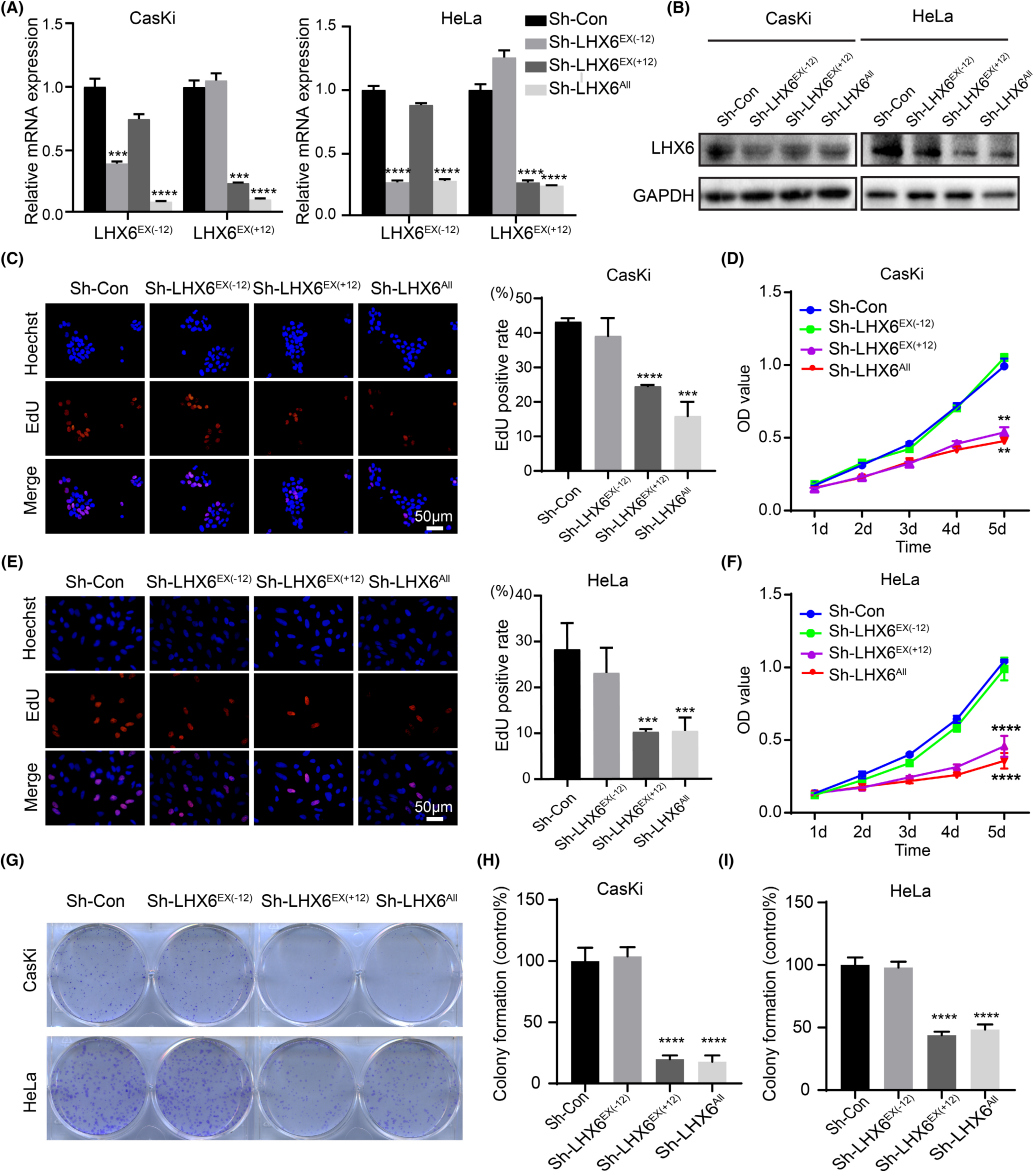
Knockdown of LHX6^EX(+12)^ and LHX6^All^ group suppressed cervical cancer cell proliferation in vitro. (A, B) ShRNA silencing of three isoform groups specifically downregulated LHX6 isoforms expression in CasKi and HeLa cells validated by qRT‐PCR (A) and western blot (B). (C, E) Cell proliferation was measured by EdU incorporation assay in CasKi (C) and HeLa cells (E). (D, F) Cell viability was measured by CCK‐8 assay in CasKi (D) and HeLa cells (F). (G‐I) Representative colony formation images and clone‐forming rate relative to the control group in CasKi and HeLa cells. The data were presented as mean ± SD (*n* = 3). **p < 0.01, ***p < 0.001, ****p < 0.0001

To explore the possible biological role of LHX6 isoforms in cervical cancer, cervical cancer cell lines CasKi and HeLa were stably transfected with short hairpin RNA (shRNA) lentivirus. Downregulation efficiency of the isoform group was verified by qRT‐PCR and western blot analysis. As seen in Figure 2A, the mRNA expression levels of LHX6^EX(−12)^ group (Sh‐LHX6^EX(−12^^)^) and LHX6^EX(+12)^ group (Sh‐LHX6^EX(+12)^) were markedly reduced compared to that of the control group (Sh‐NC), LHX6^All^ group (Sh‐LHX6^All^) downregulated the two isoform groups. In addition, the nonspecific LHX6 protein level was significantly downregulated in Sh‐LHX6^EX(+12)^ and LHX6^All^ isoform groups, but was partially reduced in Sh‐LHX6^EX(−12)^ group due to the specificity of antibody binding (Figure 2B and Figure S1). CCK‐8 methods, EdU incorporation assays and colony formation assays were performed to evaluate cell growth. Compared to the control group, silencing of the LHX6^EX(+12)^ group and LHX6^All^ group significantly suppressed cell viability, resulting in a decreased rate of EdU positive cells and a marked reduction in the number of the colony formation in CasKi and HeLa cells (Figure 2C–I). In contrast, no significant differences were found between the LHX6^EX(−12)^ knockdown group and the control group (Figure 2C–I).

**FIGURE 3:**
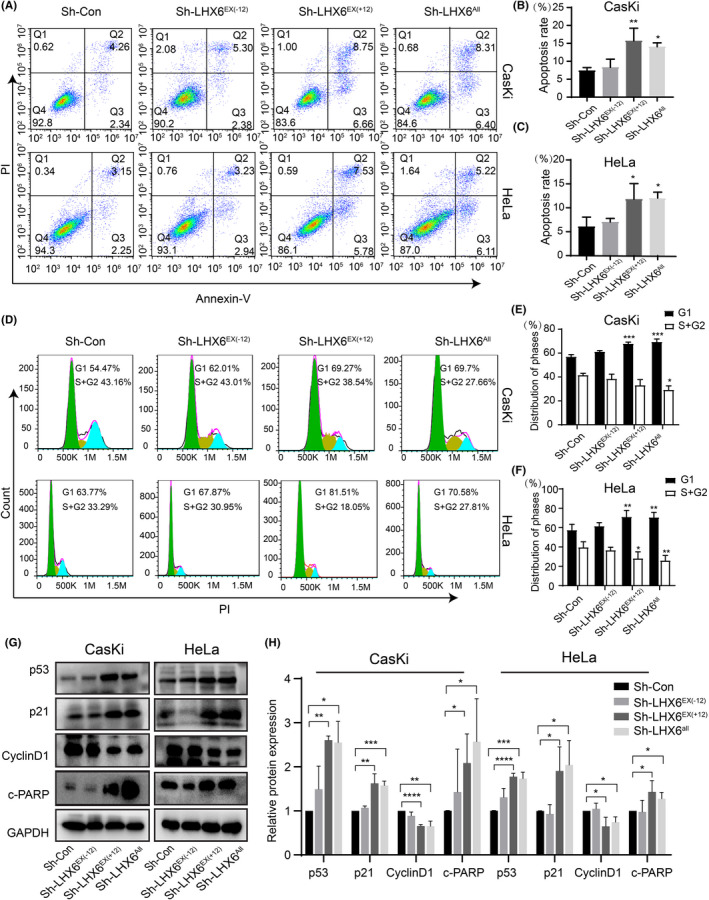
Knockdown of LHX6^EX(+12)^ and LHX6^All^ group induced cell apoptosis and cell cycle arrest in vitro. (A‐C) Cell apoptosis of CasKi and HeLa cells was detected by flow cytometry analysis. Apoptosis rate was showed in (B), (C). (D) Cell cycle of CasKi and HeLa cells were measured by flow cytometry analysis. (E, F) Distribution of different phases. (G) Cell cycle and apoptosis‐related proteins expression were detected by western blot in CasKi and HeLa cells. (H) The densitometric analyses of cell cycle and apoptosis‐related proteins in CasKi and HeLa cells. The data were presented as mean ± SD (*n* = 3). *p < 0.05, **p < 0.01, ***p < 0.001, ****p < 0.0001

In addition, we performed flow cytometry analysis to determine the apoptosis rate and cell cycle distribution in the isoform‐silenced groups and their control. As shown in Figure 3A–C, compared to the control group, knockdown of the LHX6^EX(+12)^ and LHX6^All^ group enhanced apoptosis rate (15.72 ± 3.52% vs. 14.08 ± 1.08% vs. 7.42 ± 0.81%) in CasKi cells, whereas downregulation of the LHX6^EX(−12)^ group showed no obvious changes. A similar result was found in HeLa cells (11.80 ± 3.26% vs. 12.04 ± 1.23% vs. 6.04 ± 2.02%) (Figure 3A–C). For cell cycle analysis, as shown in Figure 3D–F, knockdown of the LHX6^EX(+12)^ group and LHX6^All^ group in CasKi cells increased the percentage of cells in the G0/G1 phase (67.6 ± 1.61% vs. 69.18 ± 2.664% vs. 56.75 ± 2.025%), but reduced the percentage of cells in the S + G2/M phase (32.93 ± 4.91% vs. 29.06 ± 3.48% vs. 41.36 ± 1.73%) comparing to the control group. However, no significant differences were observed between LHX6^EX(−12)^ group and control cell. Similar cell cycle results were obtained for the HeLa cells (i.e., G0/G1 phase: 70.8 ± 6.96% vs. 70.33 ± 5.50% vs. 57.12 ± 6.33%; S + G2/M phase: 27.86 ± 7.06% vs. 25.734 ± 5.48% vs. 39.358 ± 5.99%) (Figure 3D–F). Cell apoptosis and cell cycle are regulated by a series of related proteins.^28^, ^29^ Thus, we analyzed the effects of LHX6 isoforms on the expression levels of several cell cycle and apoptosis‐related proteins. Results showed that downregulation of the LHX6^EX(+12)^ and LHX6^All^ group increased the levels of p53, p21, and cleaved‐PARP but reduced the expression of cyclinD1 in CasKi and HeLa cells (Figure 3G–H). These findings indicate that silencing of the LHX6^EX(+12)^ and LHX6^All^ group may inhibit cervical cancer cell growth by inducing cell apoptosis and cell cycle arrest. Importantly, the LHX6^EX(+12)^ isoform group may play a dominant role in the cancer‐promoting effects of LHX6 in cervical cancer.

### 3.3 Upregulation of LHX6‐4 isoform promoted cell growth, inhibited cell apoptosis, and accelerated cell cycle in cervical cancer cells in vitro

**FIGURE 4:**
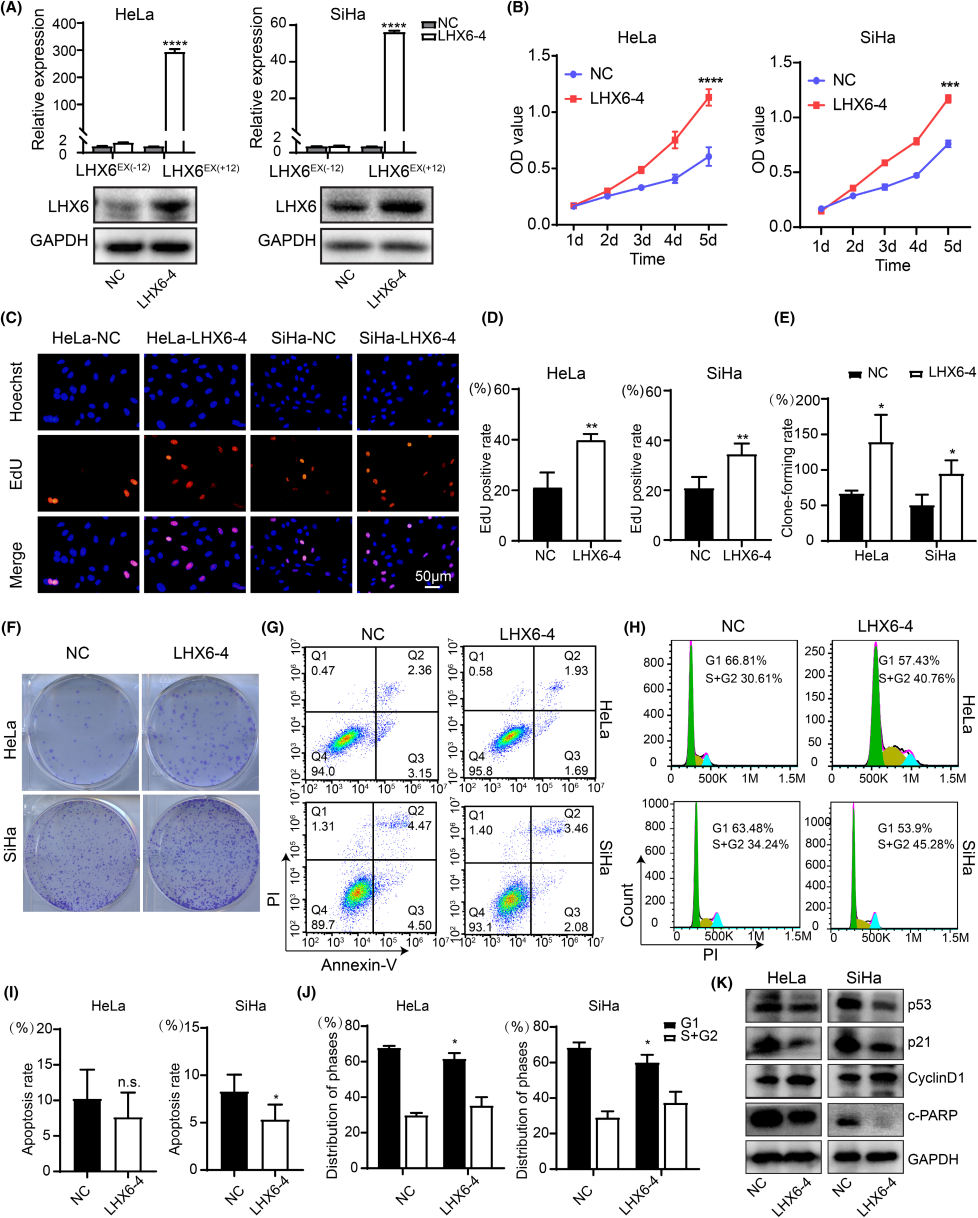
LHX6‐4 isoform overexpression promoted cell growth, inhibited cell apoptosis, and advanced cell cycle in vitro. (A) qRT‐PCR and western blot were used to verify the overexpression efficiency of LHX6‐4 isoform in HeLa and SiHa cells. (B‐F) Cell proliferation was measured by CCK‐8 (B), EdU incorporation assay (C, D) and colony formation assay (E, F) in HeLa and SiHa cells. (G‐J) Cell apoptosis and cell cycle were measured by flow cytometry. (K) Expression level of p53, p21, cyclinD1, and cleaved‐PARP were detected by western blot. The data were presented as mean ± SD (*n* = 3). *p < 0.05, **p < 0.01, ***p < 0.001, ****p < 0.0001

To further prove the involvement of the LHX6^EX(+12)^ group in the promotion of cervical cancer cell growth, we established stable LHX6^EX(+12)^ isoform‐overexpressed HeLa and SiHa cells by transfection of a lentivirus carrying the LHX6‐4 isoform. As shown in Figure 4A and Figure S2, the LHX6^EX(+12)^ group was significantly elevated in cells transfected with the LHX6‐4 isoform (referred as LHX6‐4) compared to control group (referred as NC) at both the mRNA and protein level. In agreement with our previous results, overexpression of the LHX6^EX(+12)^ group increased the cell viability, EdU positive cells and colony number in the HeLa, and SiHa cells (Figure 4B–F). As shown in Figure 4G,I, compared to the control group, overexpression of the LHX6^EX(+12)^ group resulted in a reduction in apoptotic cells in HeLa and SiHa cells (HeLa: 10.21 ± 4.12% vs.7.62 ± 3.49%; SiHa: 8.28 ± 1.79% vs. 5.32 ± 1.59%). For cell cycle analysis, as shown in Figure 4H,J, overexpression of the LHX6^EX(+12)^ group in HeLa cells reduced the percentage of cells in the G0/G1 phase (67.52 ± 1.38% vs. 61.39 ± 3.48%), but increased the percentage of cells in the S + G2/M phase (29.56 ± 1.59% vs. 35.02 ± 4.99%) comparing to the control group. Similar cell cycle results were obtained in SiHa cells (i.e., G0/G1 phase: 68.13 ± 3.15% vs. 59.71 ± 4.63%; S + G2/M phase: 28.77 ± 3.82% vs. 37.08 ± 6.48%). In addition, overexpression of the LHX6^EX(+12)^ group significantly reduced the expression levels of p53, p21, and cleaved‐PARP, but increased the expression level of cyclinD1 (Figure 4K and Figure S3). In summary, these results indicated that the LHX6^EX(+12)^ group could promote cervical cancer cell proliferation in vitro.

### 3.4 Functional analysis of the particular differentially expressed genes (DEGs) in three downregulated isoform groups

**FIGURE 5:**
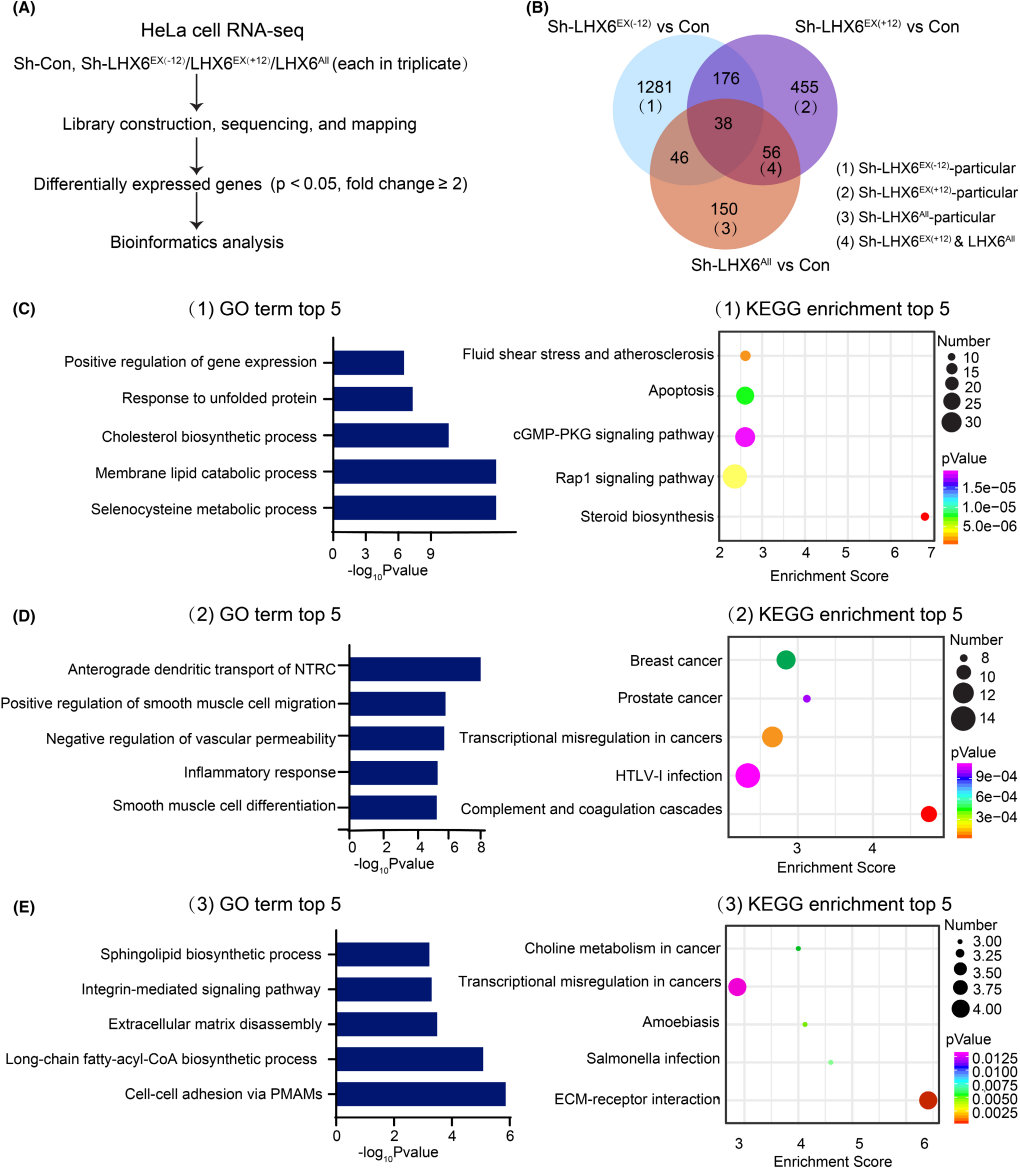
Transcriptome profiling after inhibition of three LHX6 isoform groups in HeLa cells. (A) RNA‐sequencing and analysis chart. The data were obtained from HeLa cells with specific silencing of three LHX6 isoform groups and their controls. (B) overlapped DEGs of three groups relative to their controls were shown and the four classified groups were depicted beside. (C‐E) Top 5 GO analyses terms of biological process and KEGG pathway enrichment results for the DEGs regulated by Sh‐LHX6^EX(−12)^‐particular group (C), Sh‐LHX6^EX(+12)^‐particular group (D), and Sh‐LHX6^All^‐particular group (E), respectively

To better understand the transcriptional regulatory mechanism of the LHX6 isoforms in cervical cancer, we performed RNA‐seq in the Sh‐LHX6^EX(−12)^, Sh‐LHX6^EX(+12)^, Sh‐LHX6^All^ knockdown group, and their control of HeLa cells, respectively (Figure 5A). We set screening parameters between groups (fold change ≥2 and *p* value <0.05) to acquire DEGs (Figure 5A). Compared to the control group, 1541 genes were found to be differentially expressed in the Sh‐LHX6^EX(−12)^ downregulated group, including 811 upregulated genes and 730 downregulated genes (Figure 5B and Table S2). Compared to the control group, 725 genes were significantly differentially expressed in the Sh‐LHX6^EX(+12)^ knockdown group, of which 527 genes were upregulated and 198 genes were downregulated (Figure 5B and Table S2). And compared to the control group, 290 significantly changed genes were observed in Sh‐LHX6^All^ decreased group, of which 198 genes were upregulated and 92 genes were downregulated (Figure 5B and Table S2).

To explore the differences in the transcriptional profiles of the divergent isoform groups, we classified the DEGs between the three groups and their controls into four groups: (1) particularly regulated by Sh‐LHX6^EX(−12)^ group (Sh‐LHX6^EX(−12)^‐particular); (2) particularly regulated by Sh‐LHX6^EX(+12)^ group (Sh‐LHX6^EX(+12)^‐particular); (3) particularly regulated by Sh‐LHX6^All^ group (Sh‐LHX6^All^‐particular); and (4) co‐regulated by Sh‐LHX6^EX(+12)^ group and Sh‐LHX6^All^ group but not the Sh‐LHX6^EX(−12)^ group (Sh‐LHX6^EX(+12)^ & Sh‐LHX6^All^) (Figure 5B). GO enrichment and KEGG pathway enrichment analyses were then performed to assess whether these DEGs were associated with specific biological processes and signaling pathways. For the Sh‐LHX6^EX(−12)^‐particular group, GO enrichment analysis revealed that this group may be involved in the selenocysteine metabolic process, membrane lipid catabolic process, cholesterol biosynthetic process, response to unfolded protein, and positive regulation of gene expression (Figure 5C). KEGG pathway analysis indicated that these DEGs may be related to steroid biosynthesis, Rap1 signaling pathway, and cGMP‐PKG signaling pathway (Figure 5C). For the Sh‐LHX6^EX(+12)^‐particular group, GO enrichment analysis indicated that main biological processes included anterograde dendritic transport of neurotransmitter receptor complex (NTRC), positive regulation of smooth muscle cell migration, negative regulation of vascular permeability, inflammatory response, and smooth muscle cell differentiation (Figure 5D). KEGG pathway analysis indicated the enrichment of multiple pathways, including complement and coagulation cascades, HTLV‐1 infection and transcriptional misregulation in cancers (Figure 5D). For the Sh‐LHX6^All^‐particular group, GO enrichment analysis suggested that this group was mainly involved in cell–cell adhesion via plasma‐membrane adhesion molecules (PMAMs), long‐chain fatty‐acyl‐CoA biosynthetic process, extracellular matrix disassembly, and integrin‐medicated signaling pathway (Figure 5E). The extracellular matrix (ECM)‐receptor interaction pathway was mostly enriched in the KEGG enrichment analysis (Figure 5E).

### 3.5 Downregulation of LHX6^EX^ ^(+12)^ group inhibited cervical cancer cell proliferation through regulating the MAPK signaling pathway

**FIGURE 6:**
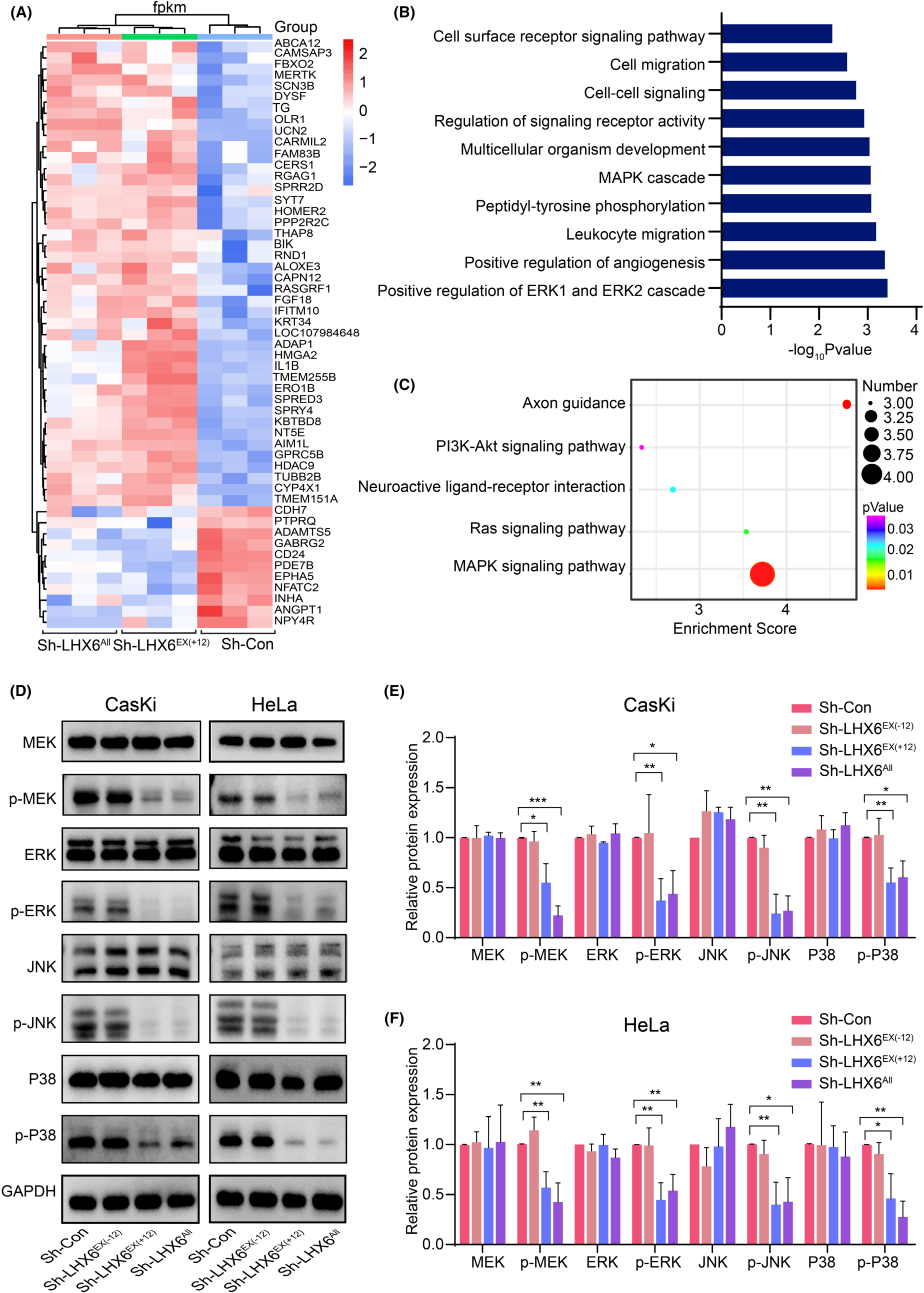
Identification of the downstream signaling pathway of LHX6^EX(+12)^ group in cervical cancer cells. (A) Heatmap of RNA‐seq data for the specific 53 genes co‐regulated by both Sh‐LHX6^EX(+12)^ group and Sh‐LHX6^All^ group but not observed in LHX6^EX(−12)^ isoform group. (B, C) Top 10 GO analyses terms of biological process and KEGG enrichment analysis results for 53 overlapped genes above. (D‐F) Western blot analysis to quantify the protein expression levels of MEK, p‐MEK, ERK, p‐ERK, JNK, p‐JNK, P38, and p‐P38 in CasKi and HeLa cells with knockdown LHX6 isoform groups

To investigate the underlying molecular mechanism related to the promotion of cervical cancer cell proliferation in the LHX6^EX(+12)^ group, bioinformatics analysis was conducted for the DEGs co‐regulated by both the Sh‐LHX6^EX(+12)^ group and Sh‐LHX6^All^ group but not were observed in the Sh‐LHX6^EX(−12)^ group (i.e., Sh‐LHX6^EX(+12)^ & Sh‐LHX6^All^ group) (Figure 5B). Interestingly, 53 out of 56 co‐regulated genes exhibited the same regulatory potential (either upregulated or downregulated), supporting our hypothesis that the LHX6^EX(+12)^ group might be the key component of LHX6 in the promotion of cervical cancer cell growth (Table S3). Among the 53 common genes, 11 genes were downregulated and 42 genes were upregulated comparing to the controls (Figure 6A and Table S3). GO enrichment analysis indicated that these genes were mainly involved in the positive regulation of ERK1 and ERK2 cascade, positive regulation of angiogenesis, MAPK cascade, cell–cell signaling, and cell migration (Figure 6B). KEGG enrichment analysis also suggested that these genes were primarily enriched in the MAPK signaling pathway (Figure 6C). These results implied that downregulation of the LHX6^EX(+12)^ group may inhibit cervical cancer cell proliferation by regulating the MAPK signaling pathway. We therefore analyzed the MAPK signaling pathway by western blot. As shown in Figure 6D‐F, compared with the control group, a reduction in the LHX6^EX(+12)^ and LHX6^All^ group significantly inhibited the phosphorylation of MEK, ERK, JNK, and P38 in CasKi and HeLa cells, although total protein levels of these genes showed no obvious changes. In contrast, no significant differences in these proteins were observed in the suppressed LHX6^EX(−12)^ group (Figure 6D–F).

## 4 DISCUSSION

Alternative splicing leads to mRNA diversity and protein complexity and maintains body homeostasis by controlling the expression of certain RNAs at specific locations at a given time. Different isoforms from a single gene can play various or even opposite roles in cancer, and thus provide potential new biomarkers and precise therapeutic targets for certain cancer.^16^, ^30^, ^31^, ^32^ LHX6 is downregulated in various cancers and inhibits cell proliferation, invasion, and migration.^23^, ^24^, ^26^, ^33^, ^34^ However, the expression patterns of different LHX6 isoforms in cervical cancer are unknown. In the current study, we found that two LHX6 isoform groups were elevated in CC samples compared to NC tissue samples, whereas the LHX6^EX(+12)^ group was observed in both NC and CC, with the positive rate of 95.24% in NC and 98.31% in CC. However, the LHX6^EX(−12)^ group could be only detected in 49.15% of CC samples. Moreover, the expression level of LHX6^EX(+12)^ group was higher than that of LHX6^EX(−12)^ group in CC tissues, which implied that LHX6 was mainly composed of LHX6^EX(+12)^ isoform group in CC samples. However, further analysis indicated that there were no significant differences between the clinicopathological parameters and the expression level of the two isoform groups in CC tissues. The results may be due to the limitation of sample size in our study, and the differential expression of the two LHX6 subtype groups may also occur in cervical cancer patients of specific subtypes or clinicopathological parameters that we do not know yet. Jung et al. had reported that two LHX6 isoforms were downregulated in cervical cancer, one named LHX6s corresponding to the LHX6 transcript variant 5 in our study.^35^ On the one hand, it was reasonable that different isoforms of a single gene may have divergent expression patterns in the same cancer type.^18^, ^36^, ^37^ On the other hand, LHX6^EX(+12)^ group in our study contained another two isoforms except for the transcript variant 5 and the two isoforms might dominate the LHX6^EX(+12)^ isoform group in cervical cancer. In addition, another isoform referred as hLHX6.1 was reported as a potential tumor suppressor gene in prior research.^38^ We noticed that the PCR primers used in that study were not specific for LHX6 to date, which may attribute to the update of human genome data.

We also described the biological role of different isoform groups in cervical cancer for the first time. Knockdown of LHX6^EX(+12)^ and LHX6^All^ group suppressed cell proliferation, induced cell apoptosis, and arrested the cell cycle, while overexpression of the LHX6‐4 isoform showed the opposite results. However, these phenomenas were not detected in the LHX6^EX(−12)^ isoform group. Cell apoptosis and cell cycle are regulated by a set of related molecules.^28^, ^29^ The above results suggested that LHX6^EX(+12)^ and LHX6^All^ isoform group may facilitate cell proliferation by inhibiting cell apoptosis and advancing the cell cycle from the G0/G1 phase to S phase. Our findings showed that the LHX6^EX(+12)^ and LHX6^All^ knockdown group induced significant changes in proteins related to cell apoptosis and cell cycle, such as upregulation of p53, p21, and cleaved‐PARP and downregulation of cyclinD1. In comparison, no apparent variances in these proteins were found in the suppressed LHX6^EX(−12)^ isoform group. Moreover, as the LHX6^EX(+12)^ isoform group was the dominant composition of LHX6, we proposed that LHX6 promoted cervical cancer cell growth mainly through the function of the LHX6^EX(+12)^ group.

RNA‐seq identified numerous DEGs between the LHX6 isoforms knockdown groups and their controls in HeLa cells. To investigate the potentially specific biological roles of the different isoform groups, further bioinformatics analysis was performed. Results showed that LHX6^EX(−12)^‐particular group was mainly involved in lipid metabolism, the LHX6^EX(+12)^‐particular group was primarily participated in cell differentiation and cell migration, and the Sh‐LHX6^All^‐particular group was mainly involved in cell–cell adhesion. However, these findings need to be verified in future investigations. As our analysis indicated that LHX6^EX(+12)^ group was the major oncogenic subtype of LHX6 in cervical cancer, hence bioinformatics analysis was proceeded for the Sh‐LHX6^EX(+12)^ & Sh‐LHX6^All^ group. GO enrichment and KEGG pathway analyses of the 53 co‐regulated genes indicated that the LHX6^EX(+12)^ isoform group may promote cervical cancer cell proliferation by regulating the MAPK signaling pathway. Classical MAPKs are comprised of four groups, that is, extracellular signal‐regulated kinases 1/2 (ERK1/2), c‐Jun amino(N)‐terminal kinases 1/2/3 (JNK1/2/3), p38, and ERK5.^39^ MAPKs play important roles in cell proliferation, differentiation, cell cycle, apoptosis, migration, and inflammation.^40^, ^41^, ^42^ Most studies suggested that increased phosphorylation of MEK, ERK, and JNK/P38 promoted cell proliferation in multiple cancers, including cervical cancer.^43^, ^44^, ^45^, ^46^, ^47^ Our study showed that knockdown of the LHX6^EX(+12)^ isoform group suppressed the phosphorylation of MEK, ERK, and JNK/P38, indicating that the LHX6^EX(+12)^ group may promote cervical cancer cell growth via regulating the MAPK signaling pathway. Few studies to date had described the role and molecular mechanism of exon 12 and its related transcripts in cancer. Exon 12 of LHX6 is composed of 104 base pairs and located on the coding area of LHX6 transcripts. As different isoforms from the mother gene could have various influence on the same cancer by binding to the specific components of downstream genes or not.^37^ We give our conjecture that exon 12‐containing LHX6 transcripts may have special mRNA sequence or encode‐specific protein regions which could bind to the components of MAPK signaling pathway or other genes that we do not know yet. And we hope that more in‐depth research could be performed on the molecular mechanism of exon 12 of LHX6 and its related transcripts in the future.

In summary, our work described, for the first time, the expression patterns and biological roles of two different LHX6 isoform groups in cervical cancer. More importantly, our results indicated that LHX6^EX(+12)^ isoform group was the dominant constituent and oncogenic type of LHX6 in cervical cancer, supporting its potentials as a new biomarker and a precise therapeutic target for cervical cancer in the future by managing the oncogenic LHX6 isoforms. Furthermore, we showed the first description that LHX6^EX(+12)^ isoform group may promote cervical cancer cell proliferation by regulating the phosphorylation of MEK, ERK, and JNK/P38 at protein level. However, the specific molecular mechanism of LHX6 exon 12 in promoting cervical cancer growth was not clearly described due to the limitation of our study. And we hope that more in‐depth research could be performed on it in the future.

## 5 CONCLUSIONS

Our study demonstrated that LHX6^EX(+12)^ and LHX6^EX(−12)^ isoform groups were differentially expressed in cervical tissues. Functional experiments and bioinformatics analyses suggested that LHX6^EX(+12)^ group promoted cervical cancer cell growth via regulating the MAPK signaling pathway. In summary, our study indicated that LHX6^EX(+12)^ isoform group was the key constituent of LHX6 in the promotion of cervical cancer cell growth, and thus may be a new biomarker and a precise therapeutic target for cervical cancer in the future.

## CONFLICT OF INTEREST

The authors declare that they have no competing interest.

## AUTHOR CONTRIBUTIONS

The study was proposed and designed by Peng Wu and Ling Xi. Clinical data collection was accomplished by Ying Zhou, Rui Wei, Guiying Jiang, Hanjie Xu, and Xueqian Wang. Cell experiments were conducted by Ling Wang, Ying Zhou, Canhui Cao, Shitong Lin, Wenhua Zhi, Danya Zhang, and Jie Li. Data analysis and interpretation were performed by Ling Wang. Ling Wang produced the manuscript which was checked by Peng Wu, Ling Xi, Ying Zhou, and Canhui Cao. All authors read and approved the final manuscript.

## CONSENT AND ETHICS APPROVAL STATEMENT

Written informed consents were obtained from patients for use of tissue samples and this study was approved by the Clinical Trial Ethics Committee of Huazhong University of Science and Technology.

## Supporting information


Table S1

Table S2

Table S3

Figures S1‐S3

## Data Availability

The data generated or analyzed during this study are available from the corresponding author upon reasonable request.
