# Supplementary material for: The exon 12‐containing LHX6 isoforms promote cervical cancer cell proliferation by regulating the MAPK signaling pathway
Source: Cancer Med. 2022 Apr 5;11(19):3657–73. doi: 10.1002/cam4.4734 (PMC9554449; doi:10.1002/cam4.4734)
Supplement: Supplementary file 4 — Figures S1‐S3 [file CAM4-11-3657-s002.docx]

**Additional file 2**

**
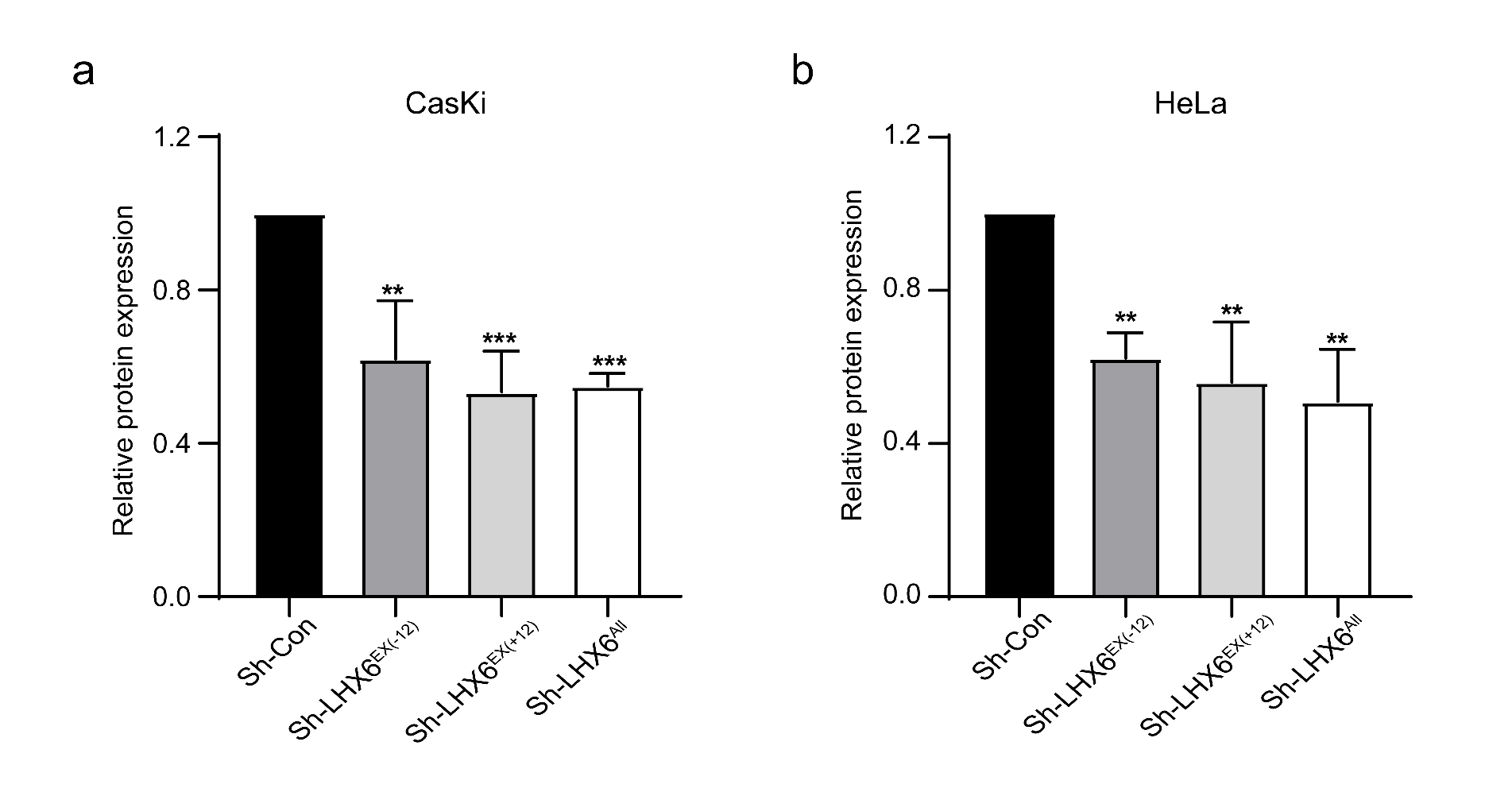
**

**Figure S1** The quantitative analyses of LHX6 protein levels in LHX6 isoform-knockdown groups. a in CasKi cells. b in HeLa cells.

**
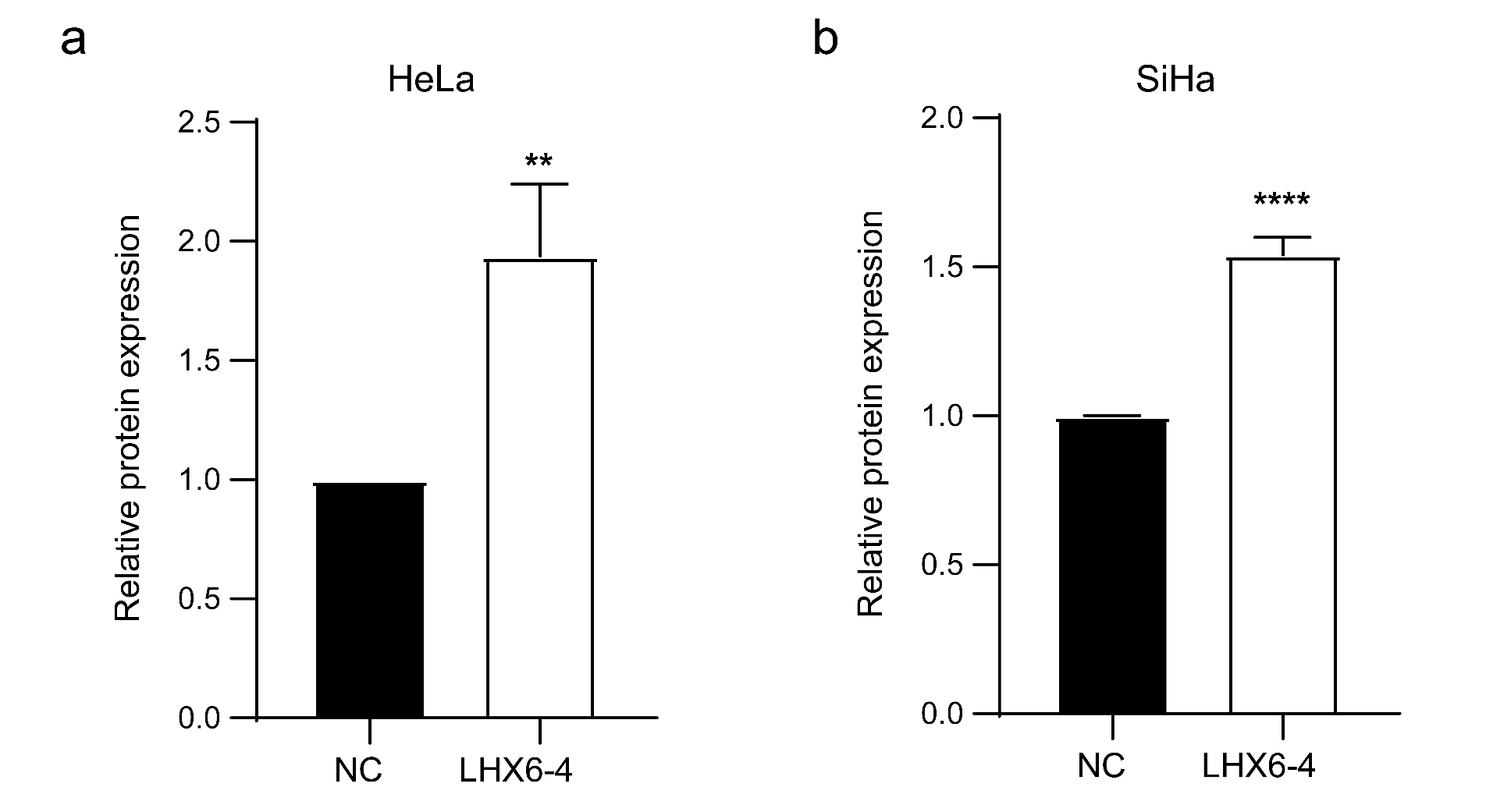
**

**Figure S2** The quantitative analyses of LHX6 protein levels in LHX6 isoform-overexpressed groups. a in HeLa cells. b in SiHa cells.


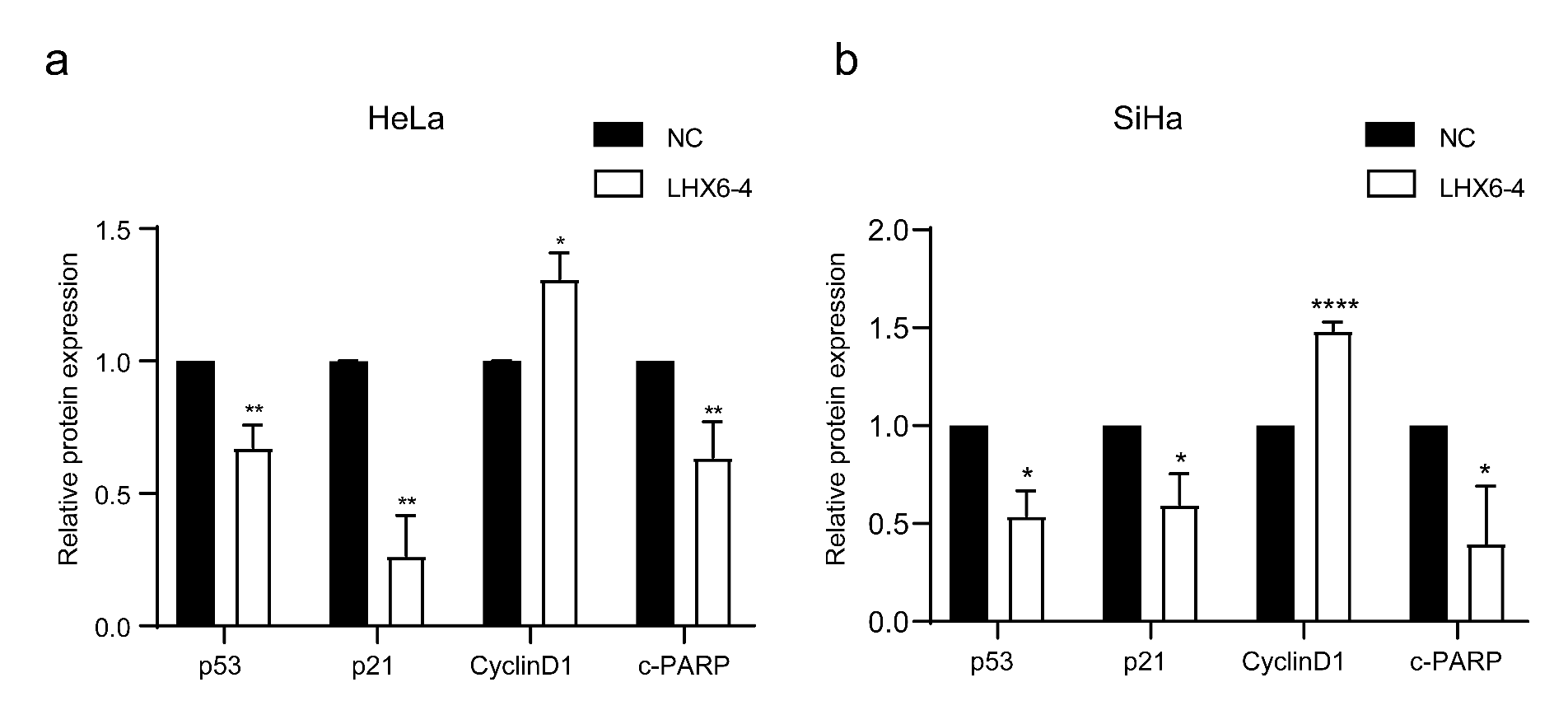


**Figure S3** The densitometric analyses of cell cycle and apoptosis-related proteins in LHX6 isoform-overexpressed groups. **a** in HeLa cells. **b** in SiHa cells.
